# Poverty and food insecurity may increase as the threat of COVID-19 spreads

**DOI:** 10.1017/S1368980020003493

**Published:** 2020-12

**Authors:** Marcos Pereira, Ana Marlucia Oliveira

**Affiliations:** 1 Institute of Collective Health, Universidade Federal da Bahia, Salvador, Brazil; 2 School of Nutrition, Universidade Federal da Bahia, Salvador, Brazil

**Keywords:** COVID, Food supply, Food insecurity, Inequality

## Abstract

This article discusses the relationship between both poverty and food insecurity (FI) and the COVID-19 pandemic, as well as presenting possible strategies and actions for increasing social protection in the fight against these conditions in the current epidemiological context, especially for low-income countries. This is a narrative review concerning COVID-19, poverty, and food and nutritional insecurity. The COVID-19 pandemic may increase poverty and FI levels, resulting from the absence of or weak political, economic and social interventions to maintain jobs, as well as compromised food production and distribution chains and reduced access to healthy foods in different countries around the world, especially the poorest ones, where social and economic inequality was already historically high; the pandemic heightens and uncovers the vulnerability of poor populations. Public policies focused on guaranteeing the human right to adequate food must be improved and implemented for populations in contexts of poverty with the aim of providing food security.

The COVID-19 pandemic raises important issues related to food and food security and living conditions in different countries. Poverty and food insecurity (FI) affect people in all regions of the world to different extents according to the availability of and access to tools that guarantee the survival of the population. The relationship between inequality and health is a field that has always been present in the daily life of marginalised populations around the world, even before the occurrence of the COVID-19. However, with the occurrence of the pandemic, an expressive portion of the population has been plunged into a state of extreme poverty, a result of the interaction between a history of structural, social and economic inequality and the expressive contingent of informal or unemployed workers, enhanced by the precarious conditions of the food production and commercialisation system, which involves agribusiness, a complex system of economic interests of large global groups^(1,2)^. These conditions are intensified by the impact of the fight for land, restricted access to drinking water and natural disasters with effects on the climate and the scarcity or lack of rain in many geographical regions of the planet^(3)^.

Within these contexts, the inadequacy of the infrastructure of society is associated with that of the life conditions of families, such as unemployment and little power to purchase goods needed for survival, including food, the lack of access to which increases in times of pandemic, plunging a large number of adults, children and the elderly around the world into poverty and FI^(4)^.

As a result of the pandemic, around 49 million people on the planet have entered into poverty conditions in 2020^(5)^. Also, the most recent estimates indicate that by the end of this year, 820 million people will suffer from hunger globally^(6)^ and more than 130 million people in the world may enter into the category of extreme hunger^(7)^.

Within this context, we will discuss how the COVID-19 pandemic may increase poverty and FI and the outlook for facing these problems, ensuring the discussion of the social, economic and political rights of the population.

Several public health questions must be currently addressed: How does FI affect the occurrence of COVID-19? What strategies can countries implement to prevent or minimise the worsening of poverty and FI during the COVID-19 pandemic?

## Relationship between COVID-19 and increased poverty and food insecurity

The COVID-19 pandemic is likely to result in even more food shortages in the world, affecting mostly underdeveloped countries. All of this is further exacerbated by recurring phenomena such as floods and droughts and by the practice of a cruel market logic, where there is an incentive to raise food prices^(4,8)^.

FI and poverty may increase dramatically in socially vulnerable populations with the expansion of the COVID-19 pandemic because it has affected income in many countries. Increased poverty and FI is expected due to the complexity of generating income in more vulnerable segments of the population, such as among informal workers, as well as the dismissal of employees from work^(4,8)^.

The reduction in the availability of food must also be considered, especially due to ruptures in the production and commercialisation chains of family agriculture in the local environment, which contributes to the onset and/or exacerbation of FI^(4,8-10)^. The closure of points of sale and commercialisation of food, for example, open markets and low-budget restaurants^(11)^, and limitations on the production of food and transportation for agricultural products may constitute disturbances for those populations that depend on local production and commercialisation and/or imports of food for family and community consumption, as well as the obtainment of income to satisfy other basic needs. Food production may also be affected by a lack of labour due to social distancing and restricted movement, as well as restrictions on imports, which could contribute to increasing FI during and after COVID-19.

People living in slums or on the streets, those deprived of freedom, as well as sex workers and people living with HIV/AIDS and other chronic diseases, are more vulnerable to FI due to biological issues or unfavourable social and economic conditions^(12-14)^. Hence, for all these groups, the risk of poverty and FI makes COVID-19 infection severe and real.

Malnutrition and moderate and severe FI can decrease the body’s immune system capacity^(15)^. In such cases, the possibility of severe complications or high mortality would increase in these groups due to infection by COVID-19. Moreover, poor immunological and virological responses may be associated with an increased risk of infectious disease transmission^(16)^.

From a biological, economic and social perspective, these groups may be undernourished and face nutritional deficiencies such as anaemia and deficiencies in vitamins A and D and antioxidant minerals (e.g. Fe, Zn and Se), which may be due to the chronic deficiencies in food consumption or impaired physiological use of nutrients^(17-19)^.

**Fig. 1:**
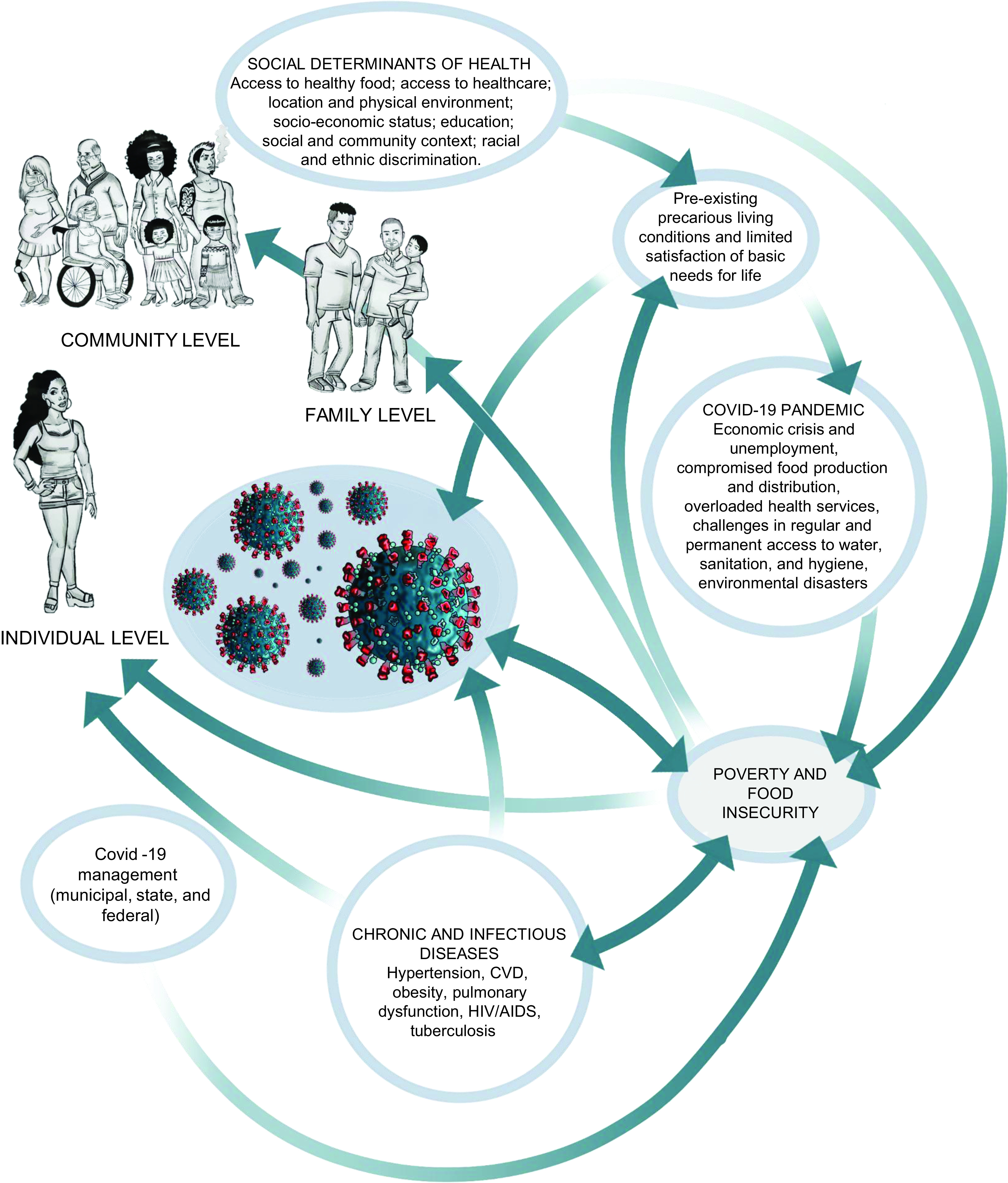
Determinants of poverty and food insecurity during the COVID-19 pandemic

The worsening of poverty and food and nutritional insecurity of a population in the pandemic context may be intensified according to the pre-existing social determinants of health, the repercussions of social policies and living conditions in the community and individual environment of indigenous, *quilombola*, black and LGBTQI+ populations, especially in poor countries where the pandemic lays bare the more perverse side of the health crisis. Within this context, it is noted that racism and discrimination, precarious socio-economic conditions and the presence of chronic diseases and previous infections can increase FI and influence COVID-19 severity (Fig. 1).

From a social protection perspective, overcoming hunger may be a challenge for controlling the COVID-19 pandemic in low-income countries. This is because people with FI may not adhere to health-protection strategies and may have difficulty following recommendations related to social isolation and hygiene. Moreover, these people may come from families where the head is unemployed or they may be informal workers living in slum agglomerations and improvised housing, sometimes with a single room and precarious personal hygiene equipment that is sometimes shared^(1)^. In addition, individuals need to work to acquire financial resources to buy food and other supplies needed for survival and are exposed to the risk of contact with the pandemic virus. Adequate social space and living conditions are challenges to be overcome in treating and preventing the occurrence of new cases of COVID-19.

Interventions in the social and economic field to overcome hunger and mitigate the impact of poverty can be a challenge for controlling the COVID-19 pandemic in low-income countries. Successful experiences in this field allow populations living in poverty and FI to overcome the social difficulties by following recommendations related to social isolation, considering the need to seek financial resources and work to buy food. Such interventions can be considered a priority in treating and preventing new cases of COVID-19.

## Reducing inequalities and choosing social protection policies for fighting food insecurity and mitigating the effect of poverty in the pandemic context

The interventions for fighting poverty and FI have been carried out in a weak and heterogeneous way in poor countries, not achieving the resolution of social inequality, even before the onset of the COVID-19 pandemic. However, in the face of the pandemic, the social movements have intensified and pressured the state to adopt economic benefit policies, particularly involving income transfers and social protection. Nonetheless, the state continues to adopt these policies as a ‘gift’ and not for intervening in social protection and taking responsibility for guaranteeing sustained access to adequate food and healthcare, which ensure social and economic protection and thus human dignity.

It is worth noting that countries with high COVID-19 mortality, such as Brazil, have seen little participation or an absence of the state in formulating policies for preventing and controlling the disease^20^, intensifying the burden of COVID-19 on populations in a context of poverty and food and nutritional security.

Incentives for the production and distribution of food to socially and economically vulnerable groups have been adopted in different countries with a view to providing food security. Financing and donations of resources to farmers’ cooperatives and small and medium businesses are being adopted to expand and improve their operations to meet the needs of local communities during the pandemic. Countries and states with policies of blocking borders and roads must enable access to urban and rural spaces with the aim of promoting the sustainability of food production and distribution.

Production and commercialisation at a national and local level must take into consideration the need to monitor food production chains and agricultural products. These strategies could be combined with tracing and monitoring job and income losses, which can negatively affect the ability of social groups to buy food and to guarantee that the food systems continue to function during the pandemic^(11)^.

Also noteworthy is the donation and distribution of personal protection equipment and disinfectants to teams at community kitchens and restaurants distributing food, with the aim of protecting workers from COVID-19 and continuing the distribution of food^(11)^.

Also observed are policies that aim to guarantee food access and financial transfers to people in contexts of vulnerability, as well as the adaptation of school food programmes, with the distribution of food/food kits and meals to students and their families. Data on food prices and stocks have also been monitored, especially in the main urban consumption centres affected by COVID-19, planning suitable actions for social, food and nutritional protection and an emergency response to increase in food prices^(11)^.

These strategies are important for mitigating social inequalities, but it is important to highlight that multibillionaires and groups with high economic power are using the pandemic as an opportunity to earn exorbitant profits with products and services^(20,21)^. Instead of allowing multibillionaires to become much richer, governments need to defend working families. Thus, it is suggested that states should tax the enormous gains that billionaires have obtained during the pandemic and use that money to guarantee healthcare and food for people and families in contexts of poverty^(20)^. Hence, it is understood that the state should assume the political, social and economic responsibility given to it, at all levels of management (municipal, state and federal).

Intervention strategies to reduce vulnerability to poverty and hunger are a duty of the state and are needed at a global level, especially in low- and middle-income countries. The COVID-19 pandemic and social isolation reduce production and food access. Therefore, strategic food storage policies at local/municipal levels should be considered to enable equitable food distribution in times of scarcity and to avoid hunger. Another possibility would be the cooperative distribution of surplus food to countries with lower agricultural production and high FI.

It is also recommended that new evidence be sought on the relationship between social vulnerability and the COVID-19 infection. In addition to strengthening existing income-transfer policies in countries, the development of food and nutrition insecurity policies and the distribution of financial resources to socially vulnerable people should be encouraged, despite the global economic crisis. In this pandemic phase, political solidarity and commitment to social welfare are expected to reduce inequalities and to guarantee the human right to adequate food and life.
